# A clinical significance analysis of manualised psychological interventions for obsessive-compulsive disorder

**DOI:** 10.1192/bjo.2021.758

**Published:** 2021-06-18

**Authors:** Jake Rigby, Peter Fisher, Gemma Cherry, Taylor Stuart, James Temple

**Affiliations:** 1 Alder Hey Children's NHS Foundation Trust; 2 University of Liverpool

## Abstract

**Aims:**

To conduct an individual patient data meta-analysis of randomised controlled trials (RCTs) of manualised psychological treatments for obsessive-compulsive disorder (OCD), and examine the differential efficacy of psychological treatments by treatment type and format.

**Background:**

Previous meta-analyses conclude that efficacious psychological treatments for OCD exist. However, determining the efficacy of psychological treatments requires multiple forms of assessment across a range of indexes, yet most previous meta-analyses in OCD are based solely on effect sizes.

**Method:**

We evaluated treatment efficacy across 24 RCTs (n = 1,667) by conducting clinical significance analyses (using standardised Jacobson methodology) and standardised mean difference within-group effect-size analyses. Outcomes were Yale-Brown Obsessive Compulsive Scale (Y-BOCS) scores, evaluated at post-treatment and follow-up (3-6 months post-treatment).

**Result:**

Post-treatment, there was a large significant within-group effect size for treated patients (g = 1.28) and a small significant effect size for controls (g = 0.30). At follow-up, large within-group effect sizes were found for both treated patients (g = 1.45) and controls (g = 0.90). Clinical significance analyses indicated that treated patients were significantly more likely than controls to recover following an intervention, but recovery rates were low; post-intervention, only 32% of treated patients and 3% of controls recovered; rising to 38% and 21% respectively at follow-up. Regardless of allocation, only approximately 20% of patients were asymptomatic at follow-up. Across the different analysis methods, individual cognitive therapy (CT) was the most effective intervention, followed by group CT plus exposure and response prevention. Self-help interventions were generally less effective.

**Conclusion:**

Reliance on aggregated within-group effect sizes may lead to overestimation of the efficacy of psychological treatments for OCD. More research is needed to determine the most effective treatment type and format for patients with OCD.

